# Occupational Highway Transportation Deaths Among Workers Aged ≥55 Years — United States, 2003–2010

**Published:** 2013-08-23

**Authors:** Stephanie G. Pratt, Rosa L. Rodríguez-Acosta

**Affiliations:** Div of Safety Research, National Institute for Occupational Safety and Health, CDC

Highway transportation incidents are the leading cause of occupational fatalities in the United States, with the highest fatality rates occurring among workers aged ≥65 years (1). To characterize older workers at highest risk, CDC analyzed data from the Census of Fatal Occupational Injuries (CFOI) for the period 2003–2010 (2) and compared occupational highway transportation deaths among workers aged 55–64 years and ≥65 years with those among workers aged 18–54 years. This report describes the results of that analysis, which indicated that workers aged ≥65 years had the highest overall fatality rate (3.1 highway transportation deaths per 100,000 full-time-equivalent [FTE] workers per year), more than three times that of workers aged 18–54 years (0.9 per 100,000 FTE workers). This pattern held across demographic and occupational categories. These results demonstrate the need to further implement interventions that consider road safety risks specific to older workers.

The U.S. Department of Labor Bureau of Labor Statistics (BLS) collects CFOI data from multiple sources. To be included in CFOI, the decedent must have been working, serving as a volunteer in a manner similar to a paid employee, or present at a site as a job requirement (2). As defined in this report, an occupational highway transportation death involved a motorized or nonmotorized vehicle and a worker aged ≥18 years, with the incident occurring on a public road, where the victim was the operator, passenger, or a pedestrian struck in or on the side of the road. Deaths while traveling between work locations are included in CFOI, whereas those during commuting to and from work are not. Fatality rates were calculated using estimates of the employed labor force from the Current Population Survey for FTE workers aged ≥18 years as denominators (3). Poisson regression methods were used to estimate fatality rates and 95% confidence intervals (CIs).

During 2003–2010, a total of 11,587 workers aged ≥18 years in the United States died in occupational highway transportation incidents, of whom 3,113 (26.9%) were aged ≥55 years. Overall, fatality rates were highest among workers aged ≥65 years (3.1 deaths per 100,000 FTE workers), followed by those aged 55–64 years (1.4 deaths per 100,000 FTE workers) (Table). Over time, fatality rates remained relatively stable for workers aged 18–54 and 55–64 years (Figure). For workers aged ≥65 years, a sharp decrease in risk was observed in 2008, but by the end of the study period, their risk for a transportation death remained more than three times the risk among those aged 18–54 years.

Risk for an occupational highway transportation death among American Indian/Alaska Native workers aged ≥65 years was more than four times the risk among those aged 18–54 years (Table). A similar pattern, although of lower magnitude, was observed among white and black workers. For Hispanic workers, the risk for an occupational highway transportation death among workers aged ≥65 years was more than twice the risk among workers aged 18–54 years; for non-Hispanic workers, the risk among workers aged ≥65 years was more than three times the risk among workers aged 18–54 years.

By primary industry, workers in transportation and warehousing accounted for a third of all deaths and had the highest rates across all age groups: 6.5, 10.6, and 21.2 for ages 18–54, 55–64, and ≥65 years, respectively (Table). By primary occupation, rates were highest in transportation and material moving occupations for all age groups: 7.4, 12.9, and 22.9 for ages 18–54, 55–64, and ≥65 years, respectively, and these occupations accounted for one half of all deaths.

The distribution of events leading to highway transportation deaths was similar across all age groups, with collisions between vehicles accounting for the largest proportion of deaths in each age group: 43%, 43%, and 48% for ages 18–54, 55–64, and ≥65 years, respectively. Across all age groups, driving a vehicle was the most common work activity being performed by the decedent. Proportions of pedestrian deaths were small: 12%–13% in all age groups. Among workers aged ≥65 years, the type of vehicle most often involved was an automobile (23%), semi-tractor trailer truck (22%), or pickup truck (15%), and a greater proportion of deaths involved off-road and industrial vehicles (9%, compared with 2% for the other age groups). Higher proportions of deaths involving semi-tractor trailer trucks were observed for workers aged 18–54 years and 55–64 years (31% and 37%, respectively).

## Editorial Note

Rates of occupational highway transportation fatalities were higher among workers aged ≥55 years compared with those aged 18–54 years, with highest rates observed among workers aged ≥65 years. In contrast, motor vehicle fatality rates for adults in the general population, including pedestrians, only begin to increase substantially at age 75 years (4).* High frequency and rates of highway transportation death for workers in transportation industries and occupations were observed in all age groups.

The safety of older workers who drive motor vehicles at work is of particular concern for employers, health professionals, and occupational safety professionals for at least four reasons. First, older workers bring a wealth of skills and experience to the workplace, making contributions beyond the traditional retirement age of 65 years. Second, starting at age 60 years, drivers involved in a crash are more likely to die from crash-related injuries than are drivers aged <60 years. This greater susceptibility to fatal injury has been found to be more important than excess crash involvement in explaining higher death rates for crash-involved drivers (5). Third, the ability to drive is affected by physical and cognitive changes associated with normal aging: declines in visual acuity, skill in processing complex visual information, reaction time, executive functioning, and contrast and glare sensitivity; and higher prevalence of comorbid conditions (6). These factors might be addressed by employers through injury prevention and wellness programs, and by workers through regular health examinations and screenings. Finally, the size of the U.S. workforce aged ≥55 years is projected to increase from approximately 15 million in 1990 to 41 million in 2020, comprising 25.2% of the workforce in 2020, compared with 11.9% in 1990 (7). Therefore, this problem is likely to increase.

Modifiable behavioral and environmental risk factors for occupational highway transportation deaths include long hours of work, fatigue, occupational stress, time pressure, distracted driving, and nonuse of seat belts (1). Interventions to mitigate these risk factors will benefit drivers of all ages. U.S. Department of Transportation regulations for drivers of large trucks and buses address many of these risk factors already.† In contrast, occupational drivers in nontransportation occupations more commonly use lighter-weight or personal vehicles such as cars and pickup trucks. Conditions of occupational use of these vehicles are largely unaddressed by occupational safety regulations issued by the federal or state governments. Additional interventions of particular benefit to all older drivers include the following: selection and adaptation of vehicles to better accommodate them; policies encouraging less driving overall, less nighttime driving, and alternative modes of transportation; route and trip planning to reduce stress and fatigue; refresher driver training; and provision of information about medical conditions and medications known to affect driving ability (8). Employers also should consider allowing drivers to use their judgment to reschedule travel or stop driving in cases of fatigue, illness, bad weather, or darkness (6). For workers who stand or walk near roadways, educational or training strategies are needed to assist older workers in compensating for age-related perceptual or cognitive deficits (9), but few evidence-based interventions are ready for employers to implement. Prevention of work-related motor vehicle crashes is a shared responsibility between employers and workers, and both groups should take an active role in developing and implementing prevention strategies.

The findings in this report are subject to at least five limitations. First, no data on nonfatal crashes and injuries were available to make comparisons with the fatality data provided by CFOI. Second, because the denominator source (the Current Population Survey) is a monthly telephone survey of households, it might underestimate the number of employed workers, especially those without permanent addresses, telephone access, or documentation of legal residency. Underestimating the employed workforce would result in overestimating the fatality rates presented in this report. Third, small numbers of deaths in some cells mean that their associated estimates of risk are unstable. Fourth, fatality rates do not account for time or distance traveled by workers in performing their jobs. Using time or distance as a rate denominator could provide different assessments of risk by employment and demographic characteristics. Finally, CFOI includes persons determined to be at work but excludes commuters. In some instances, the distinction between work travel and commuting might be unclear. Moreover, excluding commuting-related crashes underestimates the contribution of work-related travel to the overall toll of road traffic crashes.

Preventing workplace motor vehicle crashes depends on compliance with safety regulations and traffic laws, supplemented by employer-led safety initiatives and worker participation. Higher rates of highway transportation deaths for workers aged 55–64 and ≥65 years across industries and occupations support the need for employers to address specific needs and risks among older drivers in their road safety management programs and policies and in health and wellness programs. Resources are available to assist employers, health professionals, and older workers reduce the risk for motor vehicle crashes and injuries (10).

What is already known on this topic?

Highway transportation crashes are the leading cause of occupational fatalities in the United States, with the highest fatality rates occurring among workers aged ≥65 years.

What is added by this report?

Analysis of data from the Census of Fatal Occupational Injuries for the period 2003–2010 indicated that workers aged ≥65 years had the highest overall fatality rate (3.1 highway transportation deaths per 100,000 full-time-equivalent workers per year), more than three times the rate among workers aged 18–54 years. This pattern was consistent across all but one demographic group and in every industry and occupation.

What are the implications for public health practice?

Employers, health professionals, and workers need to work together to reduce risks for injury and death from highway transportation crashes among older workers. Recommended interventions to prevent crashes and injuries among older workers (e.g., trip planning, refresher driving training, and health screening and promotion) should be more widely disseminated and implemented.

## Figures and Tables

**FIGURE:**
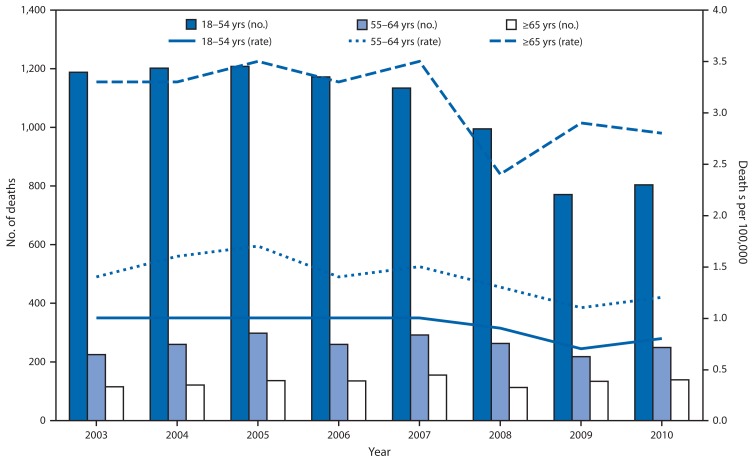
Number* and rate^†^ of occupational highway deaths, by year and age group — United States, 2003–2010 ^*^ Counts of highway transportation fatalities were generated with restricted access to the Bureau of Labor Statistics’ Census of Fatal Occupational injuries (CFOI) microdata. Fatality counts include deaths to workers aged ≥18 years, volunteer workers, and resident military personnel. Additional information is available at http://www.bls.gov/iif/oshcfoi1.htm. ^†^ Labor force denominator estimates from the Current Population Survey (CPS) for U.S. full-time equivalent (FTE) workers aged ≥18 years were used to calculate fatality rates; one FTE = 2,000 hours worked per year (additional information available at http://www.bls.gov/cps/home.htm). Volunteer workers and resident military personnel are not included in rate calculations to maintain consistency with CPS employment figures.

**TABLE:** Number* and rate† of occupational highway transportation deaths, by age group and selected characteristics — United States, 2003–2010

	Aged 18–54 yrs			Aged 55–64 yrs			Aged ≥65 yrs		
			
Characteristic	No.	Rate	(95% CI)	No.	Rate	(95% CI)	No.	Rate	(95% CI)
**Total**	**8,474**	**0.9**	**(0.9–1.0)**	**2,065**	**1.4**	**(1.3–1.4)**	**1,048**	**3.1**	**(2.9–3.3)**
**Sex**									
Male	7,677	1.5	(1.4–1.5)	1,878	2.2	(2.1–2.3)	961	4.8	(4.5–5.1)
Female	197	0.2	(0.2–0.2)	187	0.3	(0.2–0.3)	87	0.6	(0.5–0.8)
**Race**									
White	6,920	0.9	(0.9–1.0)	1,778	1.4	(1.3–1.5)	945	3.2	(3.0–3.4)
Black	1,009	1.0	(0.9–1.1)	214	1.7	(1.5–2.0)	81	3.1	(2.5–3.9)
American Indian/Alaska Native	77	1.1	(0.9–1.4)	11	1.4	(0.7–2.8)	7	4.4	(2.0–9.6)
Asian	146	0.3	(0.3–0.4)	34	0.6	(0.4–0.8)	6	0.5	(0.2–1.1)
Other	322	2.2	(2.0–2.5)	28	1.8	(1.2–2.7)	9	3.0	(1.6–5.8)
**Hispanic ethnicity**									
Hispanic	1,260	0.9	(0.9–1.0)	136	1.2	(1.0–1.5)	50	2.0	(1.5–2.7)
Non-Hispanic	7,179	0.9	(0.9–0.9)	1,919	1.4	(1.3–1.5)	993	3.2	(3.0–3.4)
**Primary industry** §									
Agriculture, forestry, fishing, and hunting	396	3.1	(2.8–3.4)	112	3.6	(2.9–4.4)	155	7.8	(6.5–9.4)
Construction	1,286	1.7	(1.7–1.9)	236	2.7	(2.3–3.1)	86	5.0	(4.0–6.2)
Leisure and hospitality	183	0.3	(0.2–0.3)	34	0.5	(0.3–0.7)	20	0.9	(0.6–1.5)
Manufacturing	364	0.3	(0.3–0.4)	108	0.6	(0.5–0.7)	40	1.5	(1.1–2.0)
Administrative support and waste management services	659	1.7	(1.6–1.9)	90	1.8	(1.4–2.2)	41	3.1	(2.3–4.3)
Mining	237	4.3	(3.8–4.9)	42	5.1	(3.7–7.1)	17	13.4	(8.2–21.9)
Other services¶	829	0.3	(0.3–0.3)	241	0.4	(0.4–0.5)	147	1.1	(0.9–1.3)
Professional, scientific, and technical services	140	0.2	(0.2–0.3)	39	0.4	(0.3–0.6)	20	0.8	(0.5–1.3)
Public administration	807	1.6	(1.4–1.7)	123	1.2	(1.0–1.4)	63	3.8	(2.8–5.0)
Retail trade	364	0.4	(0.3–0.4)	84	0.6	(0.5–0.7)	102	2.3	(1.8–2.8)
Transportation and warehousing	2,659	6.5	(6.3–6.8)	824	10.6	(9.8–11.4)	304	21.2	(18.8–23.8)
Utilities	64	0.8	(0.6–1.0)	13	0.8	(0.5–1.5)	3	1.8	(0.6–5.8)
Wholesale trade	476	1.6	(1.5–1.7)	117	2.3	(1.9–2.8)	49	4.5	(3.4–6.1)
**Primary occupation** **									
Construction and extraction	1,077	1.8	(1.7–1.9)	166	2.7	(2.3–3.2)	50	4.6	(3.4–6.1)
Farming, fishing, and forestry	196	3.0	(2.6–3.4)	38	4.5	(3.2–6.4)	21	8.1	(5.2–12.7)
Installation, maintenance, and repair	364	1.0	(0.9–1.1)	60	1.1	(0.9–1.5)	17	2.0	(1.2–3.3)
Management, business, and finance	453	0.3	(0.3–0.3)	162	0.5	(0.4–0.6)	173	2.5	(2.1–2.9)
Office and administrative	172	0.2	(0.1–0.2)	63	0.3	(0.2–0.4)	48	1.2	(0.8–1.6)
Production	115	0.2	(0.2–2.3)	24	0.2	(0.2–0.4)	16	1.0	(0.6–1.7)
Professional	171	0.3	(0.3–0.4)	37	0.5	(0.3–0.7)	8	0.7	(0.3–1.4)
Sales and related	380	0.4	(0.3–0.4)	91	0.5	(0.4–0.7)	73	1.5	(1.2–1.9)
Services	1,336	0.5	(0.5–0.5)	253	0.5	(0.5–0.6)	136	1.2	(1.0–1.5)
Transportation and material moving	4,138	7.4	(7.2–7.6)	1,166	12.9	(12.0–13.7)	505	22.9	(20.9–25.2)

**Abbreviation:** CI = confidence interval.

* Counts of highway transportation fatalities were generated with restricted access to the Bureau of Labor Statistics’ Census of Fatal Occupational injuries (CFOI) microdata. Fatality counts include deaths to workers aged ≥18 years, volunteer workers, and resident military personnel. Additional information is available at http://www.bls.gov/iif/oshcfoi1.htm.

† Rate per 100,000. Labor force denominator estimates from the Current Population Survey (CPS) for U.S. full-time equivalent (FTE) workers aged ≥18 years were used to calculate fatality rates; one FTE = 2,000 hours worked per year (additional information available at http://www.bls.gov/cps/home.htm). Volunteer workers and resident military personnel are not included in rate calculations to maintain consistency with CPS employment figures.

§ Excludes 13 fatalities with unknown primary industry.

¶ Includes education and health, finance and insurance, information, real estate, rental and leasing, and other.

** Excludes 14 fatalities with unknown primary occupation and 61 fatalities in military occupations.

## Footnotes

* Direct comparisons between occupational highway transportation fatality rates and highway transportation fatality rates in the general population cannot be made because of differences in denominator data.

† Information available at http://www.fmcsa.dot.gov.
